# Engineering of RNase P Ribozymes for Therapy against Human Cytomegalovirus Infection

**DOI:** 10.3390/v16081196

**Published:** 2024-07-25

**Authors:** Adam Smith, Isadora Zhang, Phong Trang, Fenyong Liu

**Affiliations:** 1 Program in Comparative Biochemistry, University of California, Berkeley, CA 94720, USA; 2 School of Public Health, University of California, Berkeley, CA 94720, USA

**Keywords:** ribozyme, catalytic RNA, RNase P, HCMV, antivirals, antisense, gene targeting

## Abstract

Nucleic acid-based gene interference and editing strategies, such as antisense oligonucleotides, ribozymes, RNA interference (RNAi), and CRISPR/Cas9 coupled with guide RNAs, are exciting research tools and show great promise for clinical applications in treating various illnesses. RNase P ribozymes have been engineered for therapeutic applications against human viruses such as human cytomegalovirus (HCMV). M1 ribozyme, the catalytic RNA subunit of RNase P from *Escherichia coli*, can be converted into a sequence-specific endonuclease, M1GS ribozyme, which is capable of hydrolyzing an mRNA target base-pairing with the guide sequence. M1GS RNAs have been shown to hydrolyze essential HCMV mRNAs and block viral progeny production in virus-infected cell cultures. Furthermore, RNase P ribozyme variants with enhanced hydrolyzing activity can be generated by employing in vitro selection procedures and exhibit better ability in suppressing HCMV gene expression and replication in cultured cells. Additional studies have also examined the antiviral activity of RNase P ribozymes in mice in vivo. Using cytomegalovirus infection as an example, this review summarizes the principles underlying RNase P ribozyme-mediated gene inactivation, presents recent progress in engineering RNase P ribozymes for applications in vitro and in mice, and discusses the prospects of using M1GS technology for therapeutic applications against HCMV as well as other pathogenic viruses.

## 1. Introduction

Nucleic acid-based gene interference and editing strategies, such as antisense oligonucleotides, ribozymes, RNA interference (RNAi), and CRISPR/Cas9 coupled with guide RNA, are powerful research tools and show great promise for clinical applications in treating various illnesses [1,2,3]. As a gene-targeting agent, hairpin and hammerhead ribozymes have shown exciting promise for therapeutic applications for an array of human diseases, including AIDS [4,5]. These ribozymes contain a catalytic RNA domain that cleaves the target mRNA and a substrate-binding domain with a sequence antisense to the target mRNA sequence, allowing the ribozymes to target specific sequences through Watson–Crick interactions. Various ribozymes exhibited effective gene-targeting activity in vitro and in vivo and have been explored for therapeutic applications, including therapy against human viruses such as HIV [6,7,8,9]. This review focuses on the engineering of ribonuclease P (RNase P)-based ribozymes for therapy against human cytomegalovirus (HCMV) infection.

## 2. RNase P and Its Catalytic RNA

Ribonuclease P (RNase P), a holoenzyme consisting of an RNA and several protein subunits, functions in tRNA 5′ end maturation [10,11,12]. The holoenzyme cleaves a 5′ leader sequence from tRNA precursors (pre-tRNA) and several small RNAs. The *Escherichia coli* RNase P holoenzyme has a 377 nucleotide catalytic RNA subunit known as M1 RNA and a 119 amino acid protein subunit known as C5 protein [10,11,12]. In vitro, M1 RNA alone hydrolyzes its pre-tRNA substrate under high divalent ion concentrations (e.g., 100 mM Mg^2+^) [13]. C5 protein stimulates M1 RNA cleavage at low concentrations of Mg^2+^ in vitro by stabilizing the conformation of M1 RNA and increasing interactions between the enzyme and the pre-tRNA substrate [12,14,15]. Like all bacterial protein subunits of RNase P, the C5 protein is required for RNase P activity in vivo and for the survival of the bacterial cells. In human cells, the RNase P holoenzyme, called human RNase P, is made up of an RNA subunit (H1 RNA) and at least 10 protein subunits [16,17].

Extensive investigations were conducted to elucidate the catalytic mechanism of RNase P holoenzymes and their catalytic RNAs, such as M1 RNA [10,11,12]. Tertiary interactions within the RNAs have been suggested by phylogenetic comparative analysis and mutational analysis of RNase P RNAs from different species [18,19,20]. Studies have also been performed to probe how M1 RNA interacts with C5 protein to achieve efficient cleavage [14,21]. Moreover, the three-dimensional structures of bacterial catalytic RNAs and their complexes with protein cofactors from two bacteria have been determined by crystallography and cryo-EM [22,23]. The description of the structures and functions of RNase P holoenzymes and RNase P RNAs is outside the purview of this article and can be referred to in recent reviews [12,17,24,25,26,27]. This review will primarily concentrate on gene-targeting strategies using the RNase P ribozymes and their use for therapeutic applications against HCMV infection.

## 3. RNase P Substrate Recognition and Engineering of Gene-Targeting Ribozymes from RNase P RNA

**Figure 1:**
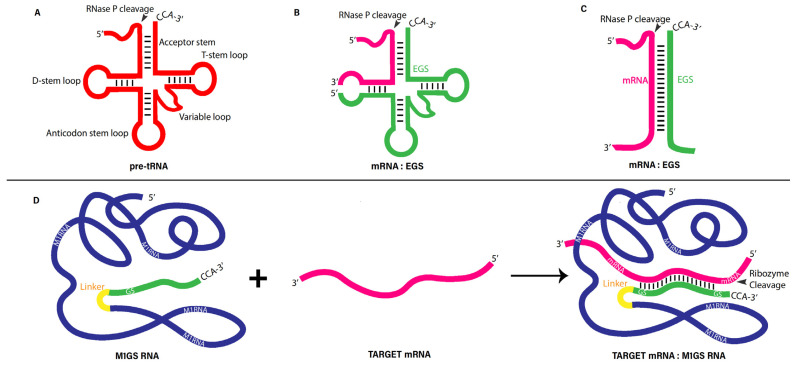
Substrates for bacterial RNase P and M1 RNA (**A**–**C**) and M1GS ribozyme binding to a target mRNA (**D**).

In *E. coli*, RNase P acts on substrates such as pre-tRNAs, the precursor to 4.5S RNA, and several small RNAs [12]. The folded structure of all these substrates is homologous to the upper part of an L-shaped pre-tRNA, with an acceptor-stem-like structure terminated with a 3′ CCA sequence and linked to a 5′ leader sequence (Figure 1A). M1 RNA was found to efficiently cleave a small model substrate with a 3′CCA sequence, 5′ leader sequence, and structures similar to the tRNA acceptor and T stems due to the base pairing of the 5′ and 3′ proximal sequences (Figure 1A) [28,29]. The 3′ proximal sequence is known as the external guide sequence (EGS) due to its ability to base pair with a target sequence in order to guide the M1 ribozyme to cleave a specific substrate (Figure 1A). EGS molecules are short antisense oligonucleotides that can modulate and knock down gene expression in bacteria and parasites [30,31,32,33,34,35] and mammalian cells [36,37] with the aid of either endogenous RNase P or M1 RNA.

Thus, EGS-based tools utilize RNase P holoenzyme or M1 RNA to hydrolyze targeted mRNAs by hybridizing the EGS with a target RNA to form a structure similar to RNase P’s tRNA substrates [28,29,37] (Figure 1A–C). Recent investigations indicated that EGS expressions in cultured mammalian cells can inhibit the expression of the EGS-targeted mRNAs and proteins and infection of human viruses, including HCMV, hepatitis B virus (HBV), HIV, influenza virus, and Kaposi sarcoma-associated herpesvirus (KSHV) [38,39,40,41,42]. Scientists also demonstrated that EGSs effectively shut down gene expression and growth in bacteria and parasites [30,31,32,33,34,35]. RNase P, in conjunction with a custom-designed EGS RNA, could, quite possibly, be used to target any RNA in any organism.

Covalently linking the EGS to M1 RNA (e.g., to the 3′ end) generates a sequence-specific ribozyme, M1GS RNA (Figure 1D), with increased cleavage efficiency and stronger substrate binding. M1GS RNA also allows more efficient cleavage relative to unlinked M1 RNA and EGSs under low Mg^2+^ concentrations [36,43,44]. So far, researchers have also demonstrated that M1GS RNAs hydrolyze many different targets, such as an oncogenic mRNA, herpes simplex virus 1 (HSV-1) essential mRNAs, and HCMV mRNAs coding, for essential functions [36,45,46,47,48,49,50].

## 4. Enhancement of M1GS through In Vitro Selection

**Figure 2:**
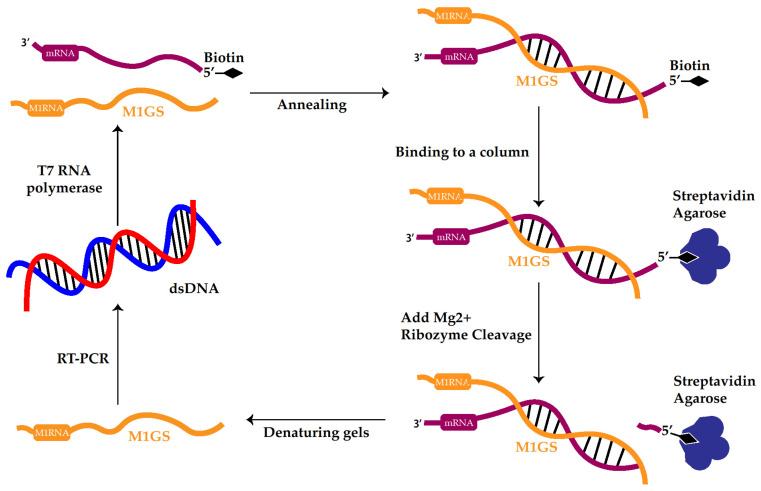
In vitro selection procedure to engineer RNase P ribozyme variants that cleave mRNA targets more efficiently.

RNase P ribozyme efficacy has been further improved in order to make it more suitable as a gene therapy tool in clinical applications. In vitro selection procedures [51,52,53] were employed to generate M1GS RNA variants that slice an mRNA substrate efficiently (Figure 2) [54,55]. For example, in one study [54], researchers generated a randomized M1 ribozyme pool with mutations in the M1 RNA catalytic and conserved regions. These mutated M1GS ribozymes were then allowed to anneal to a 5′-biotinylated RNA substrate, tk46, a model substrate sequence of the mRNA coding for HSV-1 thymidine kinase (TK) [54,55]. The annealing step was performed without Mg^2+^ ions, which is required for RNase P ribozyme-mediated hydrolysis. The annealed complexes were allowed to bind to a streptavidin column, and all unbound complexes were removed by washing the column repeatedly (Figure 2). The Mg^2+^-containing hydrolysis buffer was then added to the column to permit hydrolysis, and the active ribozymes that hydrolyzed the tk46 RNA substrate were released from the column and collected (Figure 2). The active ribozymes were purified by denaturing gel electrophoresis. DNA templates of the ribozymes were created by RT-PCR to preserve them for further rounds of selection before repeating the selection procedures until the cleavage rate ceased to increase [54]. Selection stringency was increased with each round by shortening the annealing time and incubation period. The most active ribozyme fractions were then cloned and sequenced to identify nucleotide sequence changes responsible for the enhanced activity (Figure 2) [56,57,58,59]. Many of the catalytic core mutations identified in this process were shared by multiple ribozymes. For example, multiple selected sequences (e.g., R29) contained double mutations from G_224_G_225_ to A_224_A_225_ [54]. In kinetic analyses, most of these variants exhibited an increase of at least 10-fold in cleavage of HSV-1 TK mRNA as compared to ribozymes using the wild-type M1 RNA sequence. The selected ribozymes bound to substrate tk46 with affinities (i.e., K_d(app)_ value) at least 50 times better than the wild-type ribozyme [54]. Subsequent investigations with ultraviolet crosslinking revealed that the changed nucleotide sequences and positions are close to the tk46 3′ tail sequence, implying that these changed nucleotides strengthen ribozyme–substrate interactions through direct interactions with the substrate [54,60].

## 5. M1GS Ribozymes for Studies and Treatment of HCMV Infection

A globally ubiquitous herpesvirus, HCMV, usually causes asymptomatic diseases in immunocompetent adults but results in severe morbidity or mortality in immunologically immature or immunocompromised individuals [61,62]. The virus is a leading cause of birth defects in developed countries and accounts for the majority of deaths of organ transplant patients [63,64]. It is also among the causes of opportunistic diseases in patients with AIDS, such as CMV retinitis. Current therapies against HCMV infection include antiviral drugs such as ganciclovir [61,62]. Although these drugs effectively block systemic HCMV diseases, drug resistance associated with the use of antiviral compounds has been observed and is a current concern. As such, it is crucial that novel compounds are designed, employing new mechanisms to combat HCMV infection and associated diseases.

### 5.1. M1GS-Mediated Inhibition of HCMV Infection by Targeting Viral IE1 and IE2 mRNAs

The first study applying the anti-HCMV RNase P ribozyme approach included an M1GS ribozyme targeting HCMV immediate early gene 1 and 2 (IE1/IE2) mRNAs [49]. The ribozyme was constructed to bind an overlapping exon 3 region of the mRNAs encoding IE1 and IE2, which are major HCMV transcription regulatory proteins essential for viral gene expression and HCMV progeny production [61]. A >80% decrease in expression of IE1 and IE2 was observed in human cells that stably expressed the ribozyme, along with an approximately 150-fold reduction in viral progeny production [49]. On the contrary, virus-infected cells expressing no ribozymes or a “disabled” ribozyme with changed nucleotides eliminating its hydrolysis activity showed no substantial reduction in IE1/IE2 expression (<10%) and HCMV progeny production. Human cells expressing the M1GS ribozyme also showed an overall reduction of >80% in the expression of several other HCMV late and early genes, including gH, gB, UL44, and US2. In contrast, M1GS-expressing cells did not exhibit any changes in the expression of HCMV RNAs or mRNAs that are either not under the control or modulated by IE1/IE2 (e.g., 5 kb RNA and UL36 mRNA) [49]. Thus, the antiviral effects observed were caused by the ribozyme that specifically suppressed IE1 and IE2 levels, thereby abolishing overall HCMV gene expression and progeny production.

In several follow-up studies [57,65,66], in vitro-selected M1GS ribozyme variants were also used to target the IE1 and IE2 mRNAs of HCMV. For example, a selected ribozyme variant, R27-IE, contains point mutations U_80_ -> C_80_ and C_188_ -> U_188_ in the M1 RNA sequence [66]. The R27-IE variant hydrolyzes the IE1/IE2 mRNA sequence >90 times better in vitro than the wild-type RNase P ribozyme. This variant was also significantly more effective in inhibiting viral IE1 and IE2 expression and HCMV progeny production in virus-infected cells than the wild-type ribozyme. Cells that expressed the variant showed a 99% decrease in expression of IE1 and IE2, as well as a 10,000-fold decrease in viral progeny production [66]. On the contrary, cells not expressing the ribozyme or producing a catalytically inactive ribozyme mutant showed no substantial changes in IE1/IE2 expression (<10% suppression) and viral progeny production. These studies demonstrate the effectiveness of the in vitro selection procedure in constructing gene-targeting RNase P ribozyme variants with high effectiveness in tissue culture. They also demonstrate a clear correlation between the in vitro cleavage activity (k_cat_/K_m_) of RNase P ribozymes and their efficacy in cultured cells [66]. Thus, in vitro selection may be used as a general approach to generate M1GS ribozymes with improved target mRNA cleavage efficiency and expression inhibition in cell culture.

In another study [65], a ribozyme F-R228-IE targeting the IE1 and IE2 mRNAs was derived from an M1RNA variant, R228, which exhibits three nucleotide substitution mutations (G_59_ -> A_59_, C_123_ -> U_123_, and C_326_ -> U_326_) in the catalytic domain of M1 RNA. Compared to a wild-type ribozyme, the functional ribozyme F-R228-IE hydrolyzed the target mRNAs > 100 times better in vitro. Cells with F-R228-IE expression exhibited a 98–99% suppression in IE1/IE2 levels and a 50,000-fold suppression in viral progeny production. On the contrary, cells with wild-type ribozyme F-M1-IE expression exhibited only a <80% suppression in IE1/IE2 levels and a 200-fold suppression in viral progeny production [65]. Further investigations were conducted to examine if the ribozyme also affects other aspects and steps of the viral replication cycle in cultured cells. Inhibition of the overall viral early and late gene expressions was observed as a result of the suppression of HCMV IE1 and IE2 levels by the M1GS ribozyme [65]. These observations highlighted the potential of engineered RNase P ribozymes like R27-IE and F-R228-IE, which contain new nucleotide sequence changes with better cleavage activity, as candidates for the generation of therapeutic anti-HCMV RNase P ribozyme molecules [57,65,66].

### 5.2. Studies of HCMV Protease Using the M1GS Ribozyme-Based Gene-Targeting Approach

M1GS ribozymes are also basic research tools for studying gene function by directing the guide sequence to a specific gene target. For example, M1GS ribozyme targeting the HCMV protease (PR) [61,67] was constructed to study the role of PR in viral capsid formation and its potential as a drug target [48]. Cells expressing anti-PR M1GS ribozymes demonstrated an 80% suppression in the PR level and a 100-fold suppression in HCMV progeny production after being infected with HCMV [48]. Researchers further analyzed if the anti-PR ribozymes also affect other aspects and steps of the HCMV replication cycle. These results indicated that the ribozymes had blocked viral capsid maturation and inhibited the packaging of viral DNA into the viral mature capsids.

During HCMV capsid assembly, internal scaffolding proteins are cleaved by viral PR [61,68]. In order to understand the viral capsid maturation process and the role of the PR in HCMV progeny production, ribozyme AP1 was derived from an in vitro-selected ribozyme variant, V6, that contained a C_235_ -> U_235_ substitution and insertion of an adenine immediately 5′ to C_228_ at the M1 RNA sequence [69]. A M1GS ribozyme, AP1, was constructed to target the PR mRNA. The AP1 ribozyme suppressed AP mRNA levels by >99% and led to the observation of premature PR-minus capsids in cells. Cells expressing the API ribozyme also demonstrated a 99% decrease in scaffolding protein processing and DNA encapsidation and a 10,000-fold suppression in HCMV progeny production [69]. Further study of the capsids indicated that scaffolding protein processing is independent of the capsid morphological changes during maturation. However, the protease was found to be needed for DNA encapsidation, a step essential for the production of HCMV infectious virions [69]. These studies present the protease as an excellent target for anti-HCMV therapy [48,69]. Furthermore, they demonstrate the utility of the M1GS ribozyme-based technology in studying the function of both viral and cellular genes.

### 5.3. Anti-CMV RNase P Ribozyme Studies in Mice

Several studies investigated M1GS activity in animals in vivo [70,71,72]. In one study, a functional M1GS ribozyme was designed to interact with an overlapping mRNA region of two murine cytomegalovirus (MCMV) proteins (i.e., the assembly proteins mAP and M80) [70]. The mAP and M80 proteins are involved in MCMV capsid assembly and are essential for viral progeny production. In vitro, the engineered ribozyme efficiently cleaved the target MCMV mRNAs. In cells, the ribozyme caused an 80% reduction in mAP and M80 expression and a 2000-fold suppression in MCMV progeny production. On the contrary, cells not expressing the ribozyme or producing a “disabled” ribozyme with nucleotide changes abolishing cleavage activity showed no changes in MCMV gene expression levels or progeny production. Using a modified “hydrodynamic transfection” procedure to deliver the constructs encoding the ribozymes into SCID mice, ribozyme expression was detected in the liver and spleen of MCMV-infected animals [70]. Those animals showed a significant decrease in viral gene expression levels and MCMV progeny production compared to control mice treated with disabled constructs or no constructs. Ribozyme-treated SCID mice had viral titers 200- to 2000-fold lower than control mice in the salivary glands, livers, spleens, and lungs 21 days after infection [70]. The functional ribozyme construct also significantly improved the survival rate of infected mice.

An in vitro selection procedure was used to identify new ribozyme variants with excellent activity in vitro. In another study [71], experiments were performed to investigate whether ribozyme variants selected in vitro exhibited better ability to block gene expression in animals. A new engineered ribozyme variant, R388-AS, containing three substitution mutations (G_33_ -> C_33_, C_158_ -> G_158_, and C_292_ -> A_292_) in the M1 RNA sequence, was engineered to bind to the MCMV assemblin (AS) mRNA [71]. Assemblin is an MCMV capsid protein. It is required for MCMV capsid maturation and virion formation. Compared to ribozyme M1-AS, which contained the wild-type RNase P catalytic RNA sequence, R338-AS was >200 times more efficient at hydrolyzing the mRNA target in vitro. R338-AS showed improved antiviral activity compared to the wild-type M1-AS in cultured MCMV-infected cells, exhibiting a 98–99% suppression of viral AS mRNA and protein levels and a 15,000-fold suppression of MCMV progeny generation [71]. MCMV-infected mice receiving R388-AS showed better suppression of viral AS expression levels and viral replication levels and demonstrated better animal survival than mice expressing the M1-AS ribozyme. These results demonstrated that in vitro-selected RNase P ribozyme variants with better in vitro activity were also more effective in suppressing viral gene expression in animals [70,71]. Moreover, these exciting observations implied that engineering novel RNase P ribozyme variants with nucleotide changes to improve ribozyme activity could directly increase their potential for therapeutic applications.

### 5.4. Anti-HCMV Activity of RNase P Ribozymes Delivered via Salmonella-Based Vectors

The delivery of gene-interfering agents is a fundamental challenge in gene therapy. Ideal approaches should deliver the therapeutic molecules efficiently to designated tissues/cells in a way that is tissue/cell-specific and safe. Using a differentiated macrophage model of HCMV infection, researchers employed *Salmonella*-based vectors to deliver RNase P-based ribozyme and EGS sequences to specific human cells, demonstrating ribozyme and EGS expression and viral replication suppression in these cells [72,73,74]. An M1GS ribozyme was constructed to interact with the overlapping mRNA region of HCMV capsid scaffolding protein (CSP) and assemblin. The CSP and assemblin proteins are viral capsid components, and they are required for HCMV capsid maturation and mature virion production [73]. Attenuated *Salmonella* strains that harbored the ribozyme constructs were generated and used to treat human differentiated macrophages. Considerable ribozyme expression was observed in these cells. Those cells demonstrated an 87–90% suppression in HCMV CSP levels and a 5000-fold suppression in viral progeny production as compared to cells with *Salmonella* harboring the sequence of a control ribozyme with nucleotide changes inactivating its cleavage activity [73]. These findings demonstrated that the gene transfer approach with attenuated *Salmonella* strains can be used to express ribozymes for antiviral therapeutic applications. Furthermore, these observations provide direct evidence that gene transfer of RNase P ribozymes by *Salmonella* vectors may represent a promising method for blocking HCMV infection and treating HCMV-associated diseases.

To investigate if *Salmonella* vector delivery of RNase P ribozymes is effective in blocking viral infection in vivo, a functional M1GS ribozyme was produced to interact with the overlapping mRNA region of MCMV M80.5 protein and protease [72]. M80.5 and protease are involved in MCMV capsid formation and, therefore, are required for MCMV progeny production. This was used in conjunction with a novel attenuated strain of *Salmonella* for delivery of anti-MCMV ribozymes. Attenuated *Salmonella* strains harboring the M1GS RNA constructs were generated and used to treat MCMV-infected macrophages. Cells with a functional ribozyme demonstrated an 80–85% suppression in M80.5 and protease levels and a 2500-fold suppression in viral progeny production. Mice were orally inoculated with attenuated *Salmonella* strains in order to deliver antiviral M1GS RNA to the spleen and liver. Robust ribozyme expression with no obvious adverse effects was detected in mice. In the virus-infected mice treated orally with *Salmonella* harboring the functional M1GS sequence, suppression of viral gene expression and titers, as well as enhanced survival, was observed compared to mice treated with *Salmonella* vectors alone or with *Salmonella* vectors harboring inactive control ribozymes [72]. Thus, oral delivery of M1GS RNA by attenuated *Salmonella* strains is suited to suppress viral gene expression and progeny production in mice.

## 6. Advantages and Disadvantages of M1GS Ribozymes

Classical antisense technology uses cellular RNase H to cleave an RNA-DNA hybrid to degrade the mRNA target [75,76,77]. However, this poses the risk of non-specific cleavage at non-target sites, as RNase H can act upon imperfectly hybridized RNAs [77,78]. M1GS RNA can be designed to be much more specific in hydrolyzing its targeted mRNA than the conventional antisense DNA and RNA approach [45,79]. The M1GS ribozyme can also hydrolyze its target irreversibly, and a single ribozyme molecule can hydrolyze multiple substrate molecules.

In comparison to other ribozymes, such as hairpin and hammerhead ribozymes, M1GS ribozymes possess several unique features as a gene-targeting tool. The M1GS ribozymes can fold into a defined active conformation independently of the substrates [12,79]. Also, unlike hairpin and hammerhead ribozymes, which require a specific nucleotide sequence (-GUX-) in the target mRNA, M1GS can cleave any designed sequence [4,5]. Thus, M1GS ribozymes are assumed to be capable of hydrolyzing almost any target, including stringent target regions such as the fusion junction of two chromosomes, resulting in an oncogenic chimeric mRNA [45,79]. In recent years, the RNAi approach for mRNA degradation has been developed as a promising method for nucleic acids-based therapeutic applications [80,81]. The advantages of RNAi include the ability to utilize the cellular machinery in the mRNA knockdown process and the effectiveness of the approach in vivo [82,83]. Further studies comparing the activity and effectiveness of the M1GS RNA and RNAi approaches for gene knockdown in human cells may be of interest.

It has been reported that the catalytic activity of M1RNA and M1GS ribozyme can be enhanced in the presence of human proteins, including the protein subunits/cofactors of human RNase P [36,84,85,86]. This enhancement by specific human proteins in vivo represents a unique advantage of the M1GS ribozyme approach. It is important to study the roles of these proteins in improving RNase P ribozyme targeting activity in human cells [17]. Simultaneously, it is equally important to understand potential side effects on overall cellular physiology and viability caused by interactions of overexpressed M1GS ribozymes with these proteins.

As with all gene therapy technologies, the stability and delivery of the agents remain a major concern. In order to improve stability, small ribozymes and siRNAs can be chemically synthesized with 2′ hydroxyl modifications and/or phosphorothioates to resist cellular endonucleases [83,87]. Smaller ribozymes and siRNAs can also be delivered ex vivo by encapsulating them in liposomes or other biodegradable polymeric matrices [88]. Unfortunately, M1GS’s large size (~400 nucleotides) makes the chemical synthesis of the functionally active ribozyme technically challenging. As such, endogenous and stable expression of M1GS ribozyme by viral vectors remains the most practical choice for M1GS expression and delivery.

## 7. Future Challenges and Directions

Further development of M1GS RNA for gene therapy applications will inevitably require animal models and, ultimately, human clinical trials. Pharmacokinetic and pharmacodynamic issues like ribozyme delivery, stability, colocalization, and duration of effectiveness will need to be addressed in order for M1GS RNA to be used in gene therapy against viral infections and other human diseases. For example, substantial advancements in better delivery, increased stability, and improved distribution of RNA-based therapeutics in vivo have been recently made in the mRNA vaccine field [89,90,91]. In particular, exciting research progresses have been reported on using nanoparticles for exogenous delivery of RNA molecules and introducing different modifications to the chemically or T7 RNA polymerase-synthesized RNA molecules (e.g., 1-methyl-pseudouridine) for reducing immunogenicity and immunotoxicity associated with RNA molecules [92,93]. Potentially, M1GS ribozymes synthesized with these modifications and assembled with improved nanoparticles may exhibit better pharmacokinetic properties in vivo. Further studies on these issues will facilitate the development of M1GS ribozymes for clinical applications.

HCMV, a member of the human herpesvirus family, engages in both lytic replication and latent infections [61]. The HCMV IE1 and IE2 genes encode the major viral transcription factors, and viral PR is essential for HCMV capsid formation and progeny production [61,62]. They are highly conserved among all human herpesviruses and are excellent antiviral targets for therapy against herpesviruses [94,95,96]. One avenue for the exploration of the anti-HCMV activity of M1GS RNA is the delivery of the ribozymes into hematopoietic progenitor and monocyte/macrophage-lineage cells, where HCMV is believed to establish latent infections [61]. If ribozymes can suppress the IE1/IE2 and PR expression in these cells, they may become a possible therapeutic option to prevent HCMV reactivation.

Efforts will also be needed to develop RNase P ribozymes with higher activity and better specificity for gene-targeting applications. In vitro selection procedures can be used to generate ribozyme variants that are more active and specific in knocking down gene expression. These variants also provide an added benefit in that detailed biochemical characterization can shed significant insights into the mechanisms of RNase P ribozyme cleavage of mRNA substrates and gene-targeting efficacy and specificity. Further studies of the biochemistry of RNase P ribozyme in vitro and its therapeutic activity in vivo should facilitate efforts to generate M1GS ribozymes for antiviral therapeutic applications.

## Data Availability

Not applicable.
